# Report on the Climate and Principal Diseases of the African Station

**Published:** 1847-10-01

**Authors:** 


### Report on the Climate and Principal Diseases of the African Station; Compiled from Documents in the Office of the Director-General of the Medical Department, and from other sources, in compliance with the directions of the Right Honourable the Lords Commissioners of the Admiralty. Under the immediate direction of Sir William Burnett, M.D., K.C.H., F.R.S., by *Alexander Bryson,* M.D., Surgeon R.N. 8vo. pp. 266. London, 1847.

We beg to tender our best thanks to Sir William Burnett for a copy of this most interesting and valuable Report. Would that the resources of our Army and Navy Medical Boards were more frequently brought into requisition, under the superintendence of their enlightened chiefs! That the Report before us must prove of very great utility to all medical men who may be called upon to visit the pestiferous shores of Africa, will be best appreciated by those who have been in that unhealthy region without such a companion and guide. To the readers of this Journal it cannot but be peculiarly acceptable at the present time, as forming an excellent commentary on some of the principal topics discussed in the elaborate article on Yellow Fever in our last number. All that we propose to do, just now, is to extract those portions of it which will best serve to illustrate the history of that malignant epidemic disease: we shall add but very few remarks of our own, deeming it better that each reader should investigate the points of relation between the present and the previous article for himself, and judge accordingly.

"The Topographical Remarks on the African Station, extending from Cape Verde on the North to Cape Negro on the South of the Equator," are very interesting, and form an appropriate introduction of the work. The details would, as a matter of course, be unsuitable here. We shall merely select two or three passages that will be found to bear on some of the observations and extracts in subsequent parts of our narrative.

It is well known that Sierra Leone is, perhaps, of all places along the coast—at least since Fernando Po has been abandoned—the most pernicious to the health of Europeans; at all events, the greatest mortality usually occurs there. But whether this be owing to indigenous or to accidental and contingent causes, it is not very easy to determine; seeing that there is a constant influx and reflux of strangers from different parts, and more especially the frequent arrival of prize-crews on board captured slavers. Then, too, we have to consider the extra-fatigues and exposure of the sailors, attendant upon refitting and watering there, &c.; not to mention the greater facilities which they have of committing all sorts of irregularities at this settlement than elsewhere.

"Remittent fevers occur throughout the whole year, but from November to April they are by no means of a formidable character, unless contracted under peculiarly aggravating circumstances, such as an intemperate course of living, with exposure and fatigue in the marshes, or from sleeping in the open air during the night.

"From July to October the nature of the place is totally changed, and no vessel, during that period can remain more than a week or two at a time at anchor near the settlement, with safety to the health of her crew. During the former month the rains commence, and continue to fall, with short intervals of fine weather, until September, when the river, having become enormously swollen, and of a tawny colour from the admixture of soil, overflows its banks, and floods the marshy flats behind the settlement to a great distance, where, as the rains decline and the river withdraws into its proper limits, it leaves large shallow lagoons to be evaporated by the heat of the sun and the drier winds of the succeeding months.

"During this season therefore, Europeans, whether on shore or in vessels at anchor in the river, are apt to be assailed by remitting fever of a more virulent character than during the dry season, while the more acclimated residents suffer from intermittents generally of an irregular type." P. 5.

Dr. Bryson gives a truly graphic description of the "demon of the swamp." Let it, however, be remembered, in making use of this expression, that Freetown itself "is built upon the base and side of a rocky hill, completely denuded of bush, and incapable, at any time of the year, of retaining much moisture. The hills to the westward, as far as the sea, are of the same geological formation, and are now nearly cleared of natural bush. Eastward from the town, so far as the junction of the Bunce with the main stream, there is a narrow strip of land of a more tabular form with a few patches of natural scrub, at the distance of several miles; all within that consists of cultivated fields, a race-course, villa enclosures and gardens;—yet this part of the colony has sometimes been described as a swamp, although it by no means deserves the name. Towards the eastern base of the hills, upon each side of the road to Kissy, the ground is dry, rocky, scant of soil, and, in many places, strewed over with small granitic boulders and fragments of plutonic rock. There are also several small rivulets, but nothing deserving the name of marsh. A great part of the mountain ridge behind the hill, upon which the town is built, and the intervening valley, have also been cleared of their natural thickets, whilst the latter, in many places, is under cultivation,—so that if marsh effluvia have anything to do with the constant succession of sporadic fevers occurring at Sierra Leone, which it is apprehended they have not, they must be swept by the wind from the upper part of the river at its junction with the Bunce, or from the banks of the latter, a distance, considering the astonishing elasticity and miscibility of the atmosphere, it is difficult to conceive that they could be borne without becoming thoroughly innocuous." Notwithstanding the very great difference in the physical condition of the country at different parts of the coast, the character of the prevailing and endemic diseases is the same in all.

Is it not truly melancholy to think that the fell ravages of fever are too often so powerfully aided, on the one hand, by the imprudent exposure of the crews in the boats, and, on the other, by the wicked recklessness of the men themselves when, either by permission or otherwise, they are withdrawn from the superintendence of their officers. The practice, which has been not unfrequently adopted, of increasing the quantity of grog in such a climate as that of the African Coast, cannot be over strongly reprobated. Would that, by increasing the comforts of sailors in every respect, and by the adoption of those means that are best calculated to elevate their moral character, and to make them think that they are something more than mere hired servants of the state, the use of this—often, at least—most pernicious beverage, were lessened rather than increased!

Might not the medical officers often exert a salutary influence in convincing the men of the folly and danger of unbridled indulgence? It is not by the peremptory command of a captain, so much as by the affectionate exposition of a disinterested friend, that the good object is to be attained.

We do not feel disposed to go so far as Dr. Bryson does in his, all but, condemnation of the use of tobacco. In moderation, and always provided it be not made an apology for spirit-drinking, smoking is one of the safest exhilarants in an unwholesome climate. Chewing should unquestionably be discouraged. On the whole, coffee is by far the best beverage that can be served out to the men.

It is, perhaps, scarcely necessary to preface the following observations on the medical history of the squadron on the African station from 1823 to 1845 by stating that, of all the diseases which affect the vessels there employed, Remittent fever is by far the most pernicious and destructive: "It prevails throughout the whole line of coast, at all times and seasons of the year, as an *endemic*; and, at distant and uncertain periods, it occasionally assumes an *epidemic* form."......... "In a few instances," adds Dr. Bryson, "it appears to have *acquired* contagious properties."

1823, 1824, *and* 1825.—During the month of February 1823, fever of an epidemic and malignant character began to manifest itself at Sierra

Leone, and rapidly extended to the crews of vessels in the harbour, in the river, or in the adjacent rivers and creeks, and to the different settlements along the coast. About the end of March, H.M.S. "Bann," became first affected. The subsequent history of the disease on board this ship has, it is well known, been minutely described in the admirable report of Sir William Burnett.* It will be useful to give the leading points of the narrative.

"The first case appeared on the 25th of March, after she had been at anchor off Free Town from the 11th January until that date, the crew during that time having been employed refitting the ship, and also in refitting a small prize vessel which had been converted into a tender. By the surgeon's report it also appears 'that they were much exposed to the heat of the sun's rays, and had, perhaps, indulged in irregularities,' circumstances that have never yet been known to fail in producing fever at Sierra Leone, particularly when persisted in for several weeks in succession.

"On the 26th of March the master and two seamen were next attacked, but recovered.

"On the 27th the vessel sailed from Sierra Leone, and between that date and the 31st three more cases were added to the list, and other four on the 3rd of April.

"'According to Captain Phillips' account, the sick list then rapidly increased, the disease beginning forward in the ship, came gradually to the after part, till nearly all the officers and men were attacked. Indeed, when it ceased at Ascension, about the 11th of May, only sixteen of the officers and ship's company had escaped. The total number was ninety-nine, of whom thirty-four died, fifteen of them before the Bann reached Ascension. The disease had also attacked that part of the crew which was detached in the tender, San Raphael, to reconnoitre the Gallinas; and after her return to Sierra Leone it raged with such fury that at one time it was determined to destroy her.'

"'The Bann, on her leaving Sierra Leone, was ordered to St. Thomas', but, from the unhealthy state of the crew and the bad weather, it was deemed advisable to proceed directly to the island of Ascension. On her arrival at that place, tents were erected on shore, at the distance of nearly five hundred yards from the garrison, all intercourse with which was interdicted, and the whole of the sick, amounting in number to forty-five, were landed and placed in the tents provided for them.'

"'Eighteen days after her arrival, viz., on the 11th of May, a boy (son of one of the serjeants of the garrison) was violently attacked and died, but it is neither known nor believed that he had any nearer communication with the sick of the Bann than passing daily at no great distance from the tents to feed his father's poultry.'

"About this time the fever in the Bann had nearly ceased, but it went on daily attacking some of the garrison; and it appears by the official report that twenty-eight were taken ill, of which number fifteen died and thirteen recovered. The disease finally became extinct upon the island about the 16th June." P. 37.

Sir William acknowledged his inablility to account with certainty for the origin of the fever, either in the Colony or in the Bann; but, at the same time, expressed his opinion "that it was in the first instance merely

---

* *Official Report on the Fever which appeared on board H.M. Ship Bann on the Coast of Africa, and amongst the detachment of Royal Marines, forming the Garrison on the Island of Ascension in the year* 1823. By William Burnett, M.D. &c. 8vo. pp. 78. London, 1824.

the common endemic of the country, brought on by hard labour and exposure to the sun, not possessing, under these circumstances, any contagious properties, and continued to be so until after the middle of February; that it subsequently, by the state of the weather preventing ventilation, and from a great number of the sick being confined in a small place, became contagious; and that, though it was impossible to trace the fever in question directly from the Bann to any individual of the garrison of Ascension, yet there is just reason to believe that the disease was introduced into the island by that ship."

The "Cyrene" also was at Sierra Leone in March, 1823. Several of her crew, it is stated, contracted fever from intemperance and exposure on shore. Of eleven men, who were affected, one only died; so that it is evident that the disease was not of a malignant type. During April and May, the ship was employed off the Gold Coast. Only one case of fever occurred there, in a man who slept five nights ashore in the town of Cape Coast. The Cyrene sailed for England in December. "During the year, upwards of 80 cases of fever occurred on board, principally between March and September, seven of which proved fatal."

The "Owen Glendower" arrived on the station in the early part of 1823. On the 26th of March, when at Sierra Leone, there was not a man on the sick list. Although the men were a good deal exposed, only a few cases of fever occurred. After leaving this anchorage, the duties of the crew, while employed in the boats in the Bight of Biafra, were most severe and harassing. Upwards of 70 cases of fever of a remittent character occured. "The disease," it is added, "was not contagious, and was in every instance contracted by exposure and irregularity, chiefly amongst those on detached service."

Towards the close of the year, she proceeded to Sierra Leone, where several cases of fever occurred; only one however proved fatal. In Feb. 1824, she arrived at Cape Coast, where the marines and a party of seamen were landed to garrison the castle; but, on account of the great abundance and cheapness of spirits, it was found impossible to keep the men sober. They were therefore, more particularly as their health began to suffer, re-embarked. A large proportion of them suffered subsequently from hepatitis and colonitis;—diseases which "are now of comparatively rare occurrence on this part of the coast." There occurred also a number of cases of fever, amongst the men who had been exposed at the castle.

It is scarcely necessary to detail any particulars respecting the "Swinger," or the "Maidstone." Of the former vessel we read that, when at Bunce Island, twelve miles higher up the river than Sierra Leone, "she was secured alongside the wharf, and her holds cleared out, the crew in the meantime having opportunitics of indulging immoderately in trade rum. In consequence of their imprudence, fever of a malignant character broke out and carried off eight men." It does not appear to have spread.

On board the "Victor," there occurred, between June 1824 and March 1825, between twenty and thirty cases of fever, of which five terminated fatally. In one of these fatal cases, "a liquid, like coffee-grounds, was found on dissection in the stomach."

After mentioning the cases of the "Atholl" and the "Redwing," Dr.

Bryson makes the following general remarks on the state of health during the years 1823, 1824, and 1825.

"It appears that there existed a great amount of sickness, both throughout the squadron, and throughout the different European settlements along the coast, than usually happens; but whether this resulted from accidental circumstances, or from some epidemic condition of the atmosphere, there is no means of determining. The probability however is, that both in some degree assisted in causing the more general prevalence of disease, and the increase in the mortality.

"In the first place there was a greater number of soldiers, the refuse of other regiments, sent out to Sierra Leone to fill up the ranks of the African corps, many of whom were men of incorrigibly bad habits, who in a manner drank themselves to death in a short time after they had landed in the colony.

"There was also a greater influx of Europeans upon the Gold Coast, in consequence of the Ashantee war, in which several of the vessels of the squadron also took a part, particularly in the defence of Cape Coast Castle; and it would appear that the squadron congregated more in harbour than it has ever done since. All these causes, therefore, if they did not add to the virulence of the epidemics in 1823 and in 1825, at least tended to multiply their victims.

"The prominent features of the disease, whether in the endemic or epidemic form, appear to have been identically the same with those presented to the medical officers upon the coast in the present day, notwithstanding the great diminution of bush in some parts, and particularly around Sierra Leone. Lassitude, dull erratic pains and rigors marked the stage of invasion; heat and headache, with general pains, thirst, intolerance of noise and light, irregular pyrexial exacerbations and remissions, the stage of maturation; yellowness of skin, stupor, and somnolency, dark dry tongue, irritability of stomach, black vomit, and black dejections, the stage of decline in cases terminating in death.

"In almost every instance where the disease assumed a formidable character, its origin could be traced to one or more of the common well-known predisposing and exciting causes, namely, to undue exposure to the vicissitudes of the weather, either on shore or in boats near the shore, combined with fatigue, cold, wet, insolation, or with intemperance, and other imprudences included under the head of irregularities.

"The Bann contracted the fearful scourge, which swept off nearly one-third of her crew in little more than two months, at Sierra Leone, from a protracted exposure to the influence of that pestilential locality. The Cyrene contracted a similar disease, although less virulent, from similar causes, and in the same locality. The Owen Glendower, in the Bight of Biafra, and at Sierra Leone; the Swinger, in the rivers Pongos and Bunce; the Redwing, in the rivers of Benin; and the Atholl, at Bunce Island and at Sierra Leone. It however does not appear to have assumed a contagious form in any vessel but the Bann." P. 45.

1826—1830.—It would seem that, from the year 1825 to 1829, fever did not prevail as an epidemic, or with an unusual malignancy, in any of the settlements on the coast, or on board any of the vessels of our squadron. The latter year, however, was marked by the outbreak of a most fatal pestilence, primarily (it was believed) at Sierra Leone, and subsequently at Fernando Po, and in three, if not more, of the ships of war on the station. The following details will be found to possess much interest.

The "Eden" arrived at Sierra Leone in Sept. 1827, and sailed for Fernando Po, in the beginning of October, with the necessary stores for the projected establishment at Clarence Cove on that island. In the course of the nexth month, "ulcer made its appearance on board in a somewhat malignant form." Its occurrence was attributed, no doubt very properly, "to the crowded state of the ship, there being more than double the number of people on board than there was fitting accommodation for." Notwithstanding that the vessel was several times whitewashed and otherwise purified, the disease continued (in the Spring of 1828) to prevail. It was remarked that those persons, whose ulcers became foul, were generally attacked in groups of three or four in one night, and mostly after much thunder and lightning, preceded by several days of hot, sultry, and oppressive weather. The sloughing process only attacked the lower extremities, particularly the feet and ankles, and never ascended higher than midleg.

On the 29th of October, 1827, the Eden reached her destination. It was not long before the unhealthy nature of the place became felt; several cases of fever having occurred before the end of the year. It became more general in April of the following year.

In consequence of its shewing a disposition to spread in the hospital that had been established on shore, the whole of the sick were removed on board. Besides these, two cases of fever were removed from the Horatio tender, employed in cruising off the Calabar river on the opposite coast, and one came from a prize.

During the Spring quarter, ulcer was entirely subdued; but four cases of dysentery occurred. The ship, being free from disease, sailed in June for Sierra Leone, which she reached on the 6th of July, and again left on the 21st. During this fortnight it rained with very little interruption. In the course of a few days after leaving Sierra Leone, the usual fever made its appearance, and was chiefly confined to men who had recently volunteered from timber ships, and most of whom had been living on shore and committing every kind of excess. The disease was marked by great mental depression, with but little determination to the head. The remissions were in some cases very indistinct. In the majority, there was more or less of yellow tinge in the eyes and skin; this appearance was usually first observable about the seventh day.

While at sea, between the 17th of September and the 12th of October, cruising off the Bonny and occasionally anchoring near the coast, there were not any severe cases of fever; but, on returning to Fernando Po on the day last-mentioned, five were received from the shore, in a very dangerous state. On the 20th, she sailed for the island of Ascension, having embarked seven cases of fever, all of which were contracted on shore. She returned to Fernando Po on the 26th of December, and remained (it is presumed) in Clarence Cove until the beginning of April of the next year, 1829. On the first of May she arrived at Sierra Leone, all on board being then healthy. At this time, some cases of "malignant fever" occurred on shore and on board the trading vessels in the river, *on which account medical men augured an unhealthy season.* The Eden at once began to suffer,* and from the first week in May to the 11th of June, when she arrived at Fernando Po, having left Sierra Leone on the 26th of

---

* The first cases seem to have been two midshipmen, who were taken ill on board a prize of the Eden. Both died.

May, between 40 and 50 of the crew were attacked, and of these 25 died. The whole of the officers, with the exception of one lieutenant and the gunner, were either dead, or confined to bed. "The men were dying daily, amidst almost incessant rain and frequent tornadoes, accompanied with much thunder and lightning; the main deck was crowded with sick, and constantly wet. The moral effects of these scenes became palpable in every countenance; while, from the want of medical attendance, the surgeon and two assistant-surgeons having died, it was impossible to pay that attention to the ventilation of the ship, or even to the personal comforts of the sick, which their situation required." The sick were landed on the following day upon an isolated spot; and the ship was thoroughly cleansed, whitewashed, and fumigated. But it is unnecessary to follow out the details. Suffice it to say, that "the deaths in May amounted to twenty-seven; in June, to thirty-one; in July, to thirty-two; and in August, to seven; while, out of thirty men left in hospital at Fernando Po, only nineteen were alive on the 1st of December: making the total number of deaths from fever and its sequelæ, between the 1st of May, 1829, and the 1st of December of the same year, one hundred and ten!—of whom fifty died on board the Eden, and fifty on shore at Clarence Cove. Thirteen were natives of Africa, all the others were Europeans."* The deaths must have exceeded two-thirds of the persons attacked. The disease was most indubitably malignant Yellow fever, *typhus icterodes*. The symptoms of the last stage are thus given:—"The debility increased; the eyes became more yellow, bloodshot, and glassy; the skin also became of a yellow tinge, and covered with a cold perspiration, with sordes on the teeth, chapped lips, and hurried respiration, vomiting of black matter (black vomit), sometimes delirium and convulsions; at others, coma and insensibility to surrounding objects closed the scene. All the deaths occurred between the third and ninth day of the disease, but the majority on the fourth or fifth."

Respecting the supposed origin of the pestilence, and the cause of its propagation, Dr. Bryson, after alluding to "the febrific exhalations eliminated in that pestilential spot," Sierra Leone, where the Eden was when the sickness first appeared on board, says:

"The emanations from the flats between the town and the Bunce River, and from the Bullom shore, although seven miles distant, together with the state of the weather, are therefore supposed, in the first instance, to have originated the disease, which spread with fearful rapidity and virulence over the greater part of the colony, and assumed a more than ordinary degree of malignancy amongst the shipping at anchor in front of the town; some merchant vessels in fact lost nearly all hands. From a variety of circumstances, it was considered not to have been transmissible from person to person, although it appeared to be developed in certain infected spots, and that exposure for a very short time to the exciting cause in a concentrated form, was sufficient to produce the specific effect: thus a soldier contracted fever in the Earl St. Vincent merchantman, although he remained only two hours on board." P. 65.

We must now look at the history of the "Sybille" frigate, which arrived

* "This number probably includes several men who were not entered on the ship's books." P. 64.

at Sierra Leone from England in May 1827. Four cases of remittent fever occurred on board in the beginning of June: they seem to have recovered. All that is known of the vessel for the next two years is derived from a paper, that was communicated by Dr. M'Kinnal to this Journal.* From this it appears that she had been cruising in the bights of Benin and Biafra from December 1828 to the 21st of June 1829, when she arrived at Fernando Po, the health of the crew having hitherto been wonderfully good. Here they found the "Eden," which was still suffering from the ravages of the frightful epidemic with which she had been visited. The "Champion" too was there, having arrived from Sierra Leone on the 14th of the month, *i. e.* just a week before the Sybille. The colonial surgeon and his assistant, who had come from England on board the former vessel, died soon after landing at Fernando Po.

On the 22nd, the day after her arrival, the Sybille received on board several men from the Eden. On the 23rd, one of these was attacked with fever and immediately sent on shore; and, in the course of the evening of that day, she sailed from Clarence Cove. On the 26th, a boy fell sick, and on the 2nd of July, a marine (who had come on board from the garrison of Fernando Po) was seized.

"From that period the disease continued to show itself in different parts of the ship while at sea. It soon assumed a most malignant character, and attacked individuals of every class, age, and temperament, although the negroes were affected with it in comparatively small numbers, and in a mild degree. On the most minute investigation, the surgeon was unable to trace the disease, either from man to man, or from mess to mess; and those who attended the sick were not more affected than those who kept aloof. The sick-berth attendant, and the surgeon himself, though both unprotected by previous attacks, escaped the fever. The sailmaker, who sewed up the dead bodies in their hammocks, had a slight attack, though he had formerly had the yellow fever at Jamaica; while the boy who assisted him escaped. Every attention was paid to cleanliness and ventilation; and the dead bodies were speedily committed to the deep, together with their bedding and clothes.

"'The disease was evidently yellow fever in the greatest degree of intensity. With two exceptions, it was of the continued kind; the stage of excitement short. In the worst cases, it terminated fatally between the third and sixth day, most frequently on the fifth. Death was preceded in a great number of cases by black vomit, often accompanied by a dingy or livid hue of the countenance. Yellowness of the eyes and skin was very common before death; it varied from a pale lemon colour, to a dark orange hue. An officer, who died on the eleventh day of a relapse, had previously suffered from yellow fever in the West Indies.'

"It is worthy of remark, that of the eight marines received from Fernando Po and the Eden, who took bark during eight days, two only became affected with fever.

"'The sudden cessation of the disease,' says Dr. M'Kinnal, 'on the 28th of August, when forty men were on the list, and when the power of contagion (if it existed) must have been at its height, seems to prove that atmospheric changes had great influence in the production of the disease, as well as in its extinction.'

"'On the 12th of September, the ship arrived at St. Helena without a man on the sick list; being the seventeenth day from the date of the last seizure, and the third day from the date of the last death by fever. After two days' quarantine,

* Vide Medico-Chirurgical Review for October 1830.

the officers and men of the Sybille went on shore, and mixed with the inhabitants from that time till the 25th of October, without any accident resulting from the intercourse.' " P. 54.

The Sybille again anchored at Fernando Po in November, when the settlement was considered healthy. She touched at Princes' Island, and on the 5th and 6th of December was at anchor off Whydah. She then sailed on a cruise, and arrived at Princes' Island on the 3rd of January 1830, all well on board. There she was joined by her tender, the " Black Joke," which arrived from Sierra Leone, where she had been very sickly and lost 23 of her men. Her crew, however, were now entirely recovered or convalescent.

" When the Sybille was weighing anchor on the 7th, a boy, who laboured under common tertian, came on board from the Tyne, which latter vessel was stated to be healthy. Six days afterwards, namely, on the 13th of January, yellow fever again broke out in the Sybille, while cruising off Cape Formosa. Dr. M'Kechnie, an assistant-surgeon, who had lately come from England, and who had been on board the Black Joke for a few minutes, was the first seized. The disease soon increased in the ship, and early in February it became very alarming, producing the most dreadful havoc amongst all classes on board. The number of cases during this visitation amounted to eighty-seven; of which twenty-six died, with the usual symptoms of the most malignant yellow fever."

" Afterwards, in St. Helena roads, on the 22nd of March, the fever again broke out, twenty-two cases occurred, and six died." P. 54.

After this period, there does not appear to have been any malignant fever in the Sybille.

The circumstances connected with these two destructive attacks of the disease on board—the first having occurred immediately after communicating with the " Eden," which had lost her captain, surgeon, assistant-surgeon, and a great many men by fever contracted at Sierra Leone, and the second after communication with the " Black Joke"—reasonably suggest the idea of infection as the cause of the sickness. The surgeon, indeed, attributed it principally to " noxious emanations from the interior of the ship, probably caused by the decomposition of the wood from the long-continued action of heat and moisture, aided perhaps by an accumulation of different substances under the limber-boards of the holds." But then, the existence of such an accumulation has been positively denied by officers who served in the ship, and who have asserted that no vessel could be cleaner or better ventilated. Lastly, we must not omit to mention that, although her surgeon uniformly maintained that the fever was not infectious, the officers and men thought it was highly so. The commodore, from humane motives, prohibited all Europeans, with the exception of himself and the medical officers, from visiting the sick.

We have now a few words to say respecting the Medical History of Fernando Po. That remittent fever is *endemic,* and therefore continually present there, has been too well known ever since the place was first occupied in Oct. 1827; but it was not until the end of June 1829, that it prevailed *epidemically* in the settlement. It has been seen that the Eden arrived there from Sierra Leone in a most sickly state on the 11th of this month; and, after a few days quarantine, was permitted to send her sick on shore; that the Champion arrived from the same place on the 14th, and that several bad cases of fever had been landed from her; and that the Sybille arrived on the 21st, her crew at that time in health: her subsequent history has just been given. Now for the outbreak of the epidemic fever on the island:

"On the 29th of June, a serjeant of marines was attacked; on the following day four other cases occurred, and it then became general; between the above date and the 31st of August, there were no less than seventy-seven persons prostrated by the disease; to thirty-nine of whom it proved fatal, a mortality that sufficiently stamps the malignancy of the disease. It however appears that the season had been unusually wet during the months of July and August, when the disease had acquired its greatest virulence." P. 68.

It deserves, however, to be stated that Fernando Po is, in all years, and especially at certain seasons, exceedingly unhealthy. As a proof of this, it may be stated that "of thirty mechanics, who arrived there in November 1837, all had suffered; the number that died cannot be ascertained; a few were invalided, and five only remained when the Eden arrived in June 1828;" and that "of the whole party of marines and mechanics, including officers and women, landed upon the island in the middle of June, amounting to fifty-eight, only four had escaped an attack of fever on the 31st October, 1829, and most of the others had experienced two." Altogether, Fernando Po is unquestionably one of the most pestiferous and deadly spots on the face of the wide globe. After about five years' occupation, it was at length utterly abandoned in the beginning of 1833. It should be stated that, while the epidemic of 1829 was raging, several cases occurred of invalids being received from shore on board ships in the harbour, without communicating the disease to the rest of the crew. We must not omit to mention, at the same time, that "this fever was distinctly remittent in its character, and accompanied by yellow suffusion of the skin and eyes, and by black vomit."

Dr. Bryson, after carefully considering all the circumstances of the case, makes the following significant remarks:

"It will be observed that both the Eden and Champion contracted the disease at this colony (Sierra Leone), and in their crowded state carried it with them to Fernando Po, where the supernumeraries for the colony and all the sick were landed in the course of a few days after their arrival. Amongst the former it continued to rage for the two succeeding months with unparalleled fury, and also assailed other individuals who had no direct communication with either vessel. It also appears that it broke out in the Hecla at Sierra Leone, or shortly after she left it, and carried off thirty-nine of her ship's company. The Sybille, as previously detailed, contracted the disease at Fernando Po in June, and in the course of a few weeks lost twenty-two men out of the sixty-nine attacked. In the Black Joke, however, in prizes, and otherwise, she lost in addition thirty-seven, making the total number of deaths fifty-nine for the year. There was not any vessel that suffered in the same proportion with the Eden; during the year, out of a crew it is supposed of not more than one hundred and sixty men, she lost altogether ninety-nine, sixty of whom died on board, and thirty-nine on shore. The loss in all the other vessels on the station during the year was, comparatively speaking, trifling, with the exception of the Sybille's tender, the Black Joke, from which there was not any returns sent into office in consequence of her being attached to that vessel, and manned exclusively out of her ship's company; it is, however, ascertained that she also contracted the disease at Sierra Leone.

"After the disease had been extinct for a period of upwards of four months it reappeared in the Sybille in January, 1830, and again, in the same ship, at St. Helena in March. In these two visitations her losses amounted to thirty-two men, and then it may be said the epidemic of 1829 and 1830 finally ceased, although fever of an equally virulent character nearly unmanned the Plumper in the November and December of the latter year at Sierra Leone." P. 87.

*From* 1831 *to* 1836.—During the five years following 1830, fever in a malignant and epidemic form did not appear in any of the vessels of the squadron, the "Conflict" excepted. For twelve months after her arrival on the station, she suffered but little from the common remittent of the country; but in July 1831, after the greater part of the crew had been on shore at Sierra Leone and allowed to commit the greatest excesses, thirty cases of bad fever, of which eight terminated fatally on board and five were sent to the hospital, were the result of these imprudences.

"The disease appears to have been of a most malignant character. It was remittent, but varied in its symptoms in different cases; in the worst it was attended with great excitement, and, as it advanced, the skin assumed a yellow colour, interspersed with livid spots. Towards dissolution, in the fatal cases, a quantity of dark matter was vomited, while a disagreeable cadaverous smell exhaled from the body some hours before life became extinct. The dejections were also frequently dark and fetid. In one instance, in the course of half an hour after death, the whole surface of the body presented a dark blue colour, and the cuticle separated. There can be but little doubt that all these cases were what is usually termed yellow fever in its worst form. Still, although every circumstance is very minutely detailed, contagion is not alluded to." P. 96.

The Conflict sailed in August for Ascension, where, upon being overhauled, "the hold presented, on the removal of the tanks and limberboards, a very filthy appearance, blackish mud with vegetable matter being brought into view, the effluvium from which was at first insufferable. The passages to the pump-well were found to be completely blocked up."

1837 *and* 1838.—The "Ætna" returned from Gibraltar to the African station in Nov. 1837, and anchored at Sierra Leone on the 30th, all on board being then quite well. Malignant fever was at the time committing great ravages on shore, and amongst the shipping. The Ætna only remained until the 3rd of December for the purpose of watering, and this was effected by the Kroomen: she then proceeded to sea. The weather was still calm, wet and sultry.

"On the 10th of the month two cases of fever occurred, and on the 12th there were two more; of these three died, one on the fifth day, and two on the seventh day of the disease, with black vomit and yellowness of the skin. There were not any fresh attacks until the 20th of December, when two others occurred, and on the 21st there were five. The disease then began to attack officers and men indiscriminately. As it was considered to be contagious, recourse was had to artificial means of ventilation, by swinging stoves and windsails, and to fumigation, by whitewashing the decks and sprinkling them with chloride of lime. In the meantime a course was shaped for the region of the trade winds, with the view of making Ascension; on getting into the S. E. trade however, on the 15th of January, the violence of the disease did not abate, but on the contrary, it continued to attack one after another of the remaining few who had hitherto escaped with as much virulence as it did when the ship was becalmed in the immediate neighbourhood of the land; nor did it entirely cease until the 20th of

January, the day on which she anchored at the above island. The total number attacked was ninety-nine, including one Krooman and four African boys; of these twenty-five died. The total number of the ship's company, exclusive of the Africans, was ninety-eight, and of these only five escaped, two of the latter being nearly all the time on the sick list, one with intermittent fever, and the other with rheumatism.

"The disease was considered to have been contracted at Sierra Leone, and its influence was supposed to have been the greater upon the ship's company from mental depression, in consequence of their being obliged to return to the coast of Africa instead of being paid off, as they had anticipated, from a general scorbutic taint amongst them at the time, from the laborious nature of the service on which they were employed, and from incidental privations peculiar to it. The fever was distinctly of a remitting character, attended with yellow suffusion of the skin and eyes, hæmorrhage from the gums and fauces, and, in the fatal cases, with black vamit." P. 120.

No allusion is made as whether any, and what, measures were taken at Ascension, on the arrival of the ship.

It is worthy of notice that the Ætna returned to the coast towards the end of spring and re-commenced surveying, the convalescents having been discharged to duty, although still in a weakly state. Upon the whole, however, the crew continued tolerably healthy until she returned to England in October 1838.

The "Bonetta" and the "Forester" were also very sickly at the beginning of this year, 1838. The former left the coast (in a healthy state we presume, for nothing is said to the contrary), having taken on board a supply of Indian corn and yams for the island of Ascension. In the second week of January, she fell in with the latter in a sickly state, several fatal cases of bad fever having occurred on board. After receiving a prize-crew from her, the vessels separated; and the Bonetta proceeded on her voyage to Ascension, which she reached on the 20th January, having lost eight men from the disease on the passage. The report continues thus :—

"In consequence of the illness and death of the assistant-surgeon, there is not any account of the origin and progress of the disease until another joined on the 3rd of February, when the state of the sick list and ship were as follows :—The commander, master, assistant-surgeon, purser, and twenty-eight seamen and marines, were all lying about the deck in a most helpless and melancholy state, three with black vomit, and to all appearance beyond the aid of medicine. The vessel was in a very filthy condition, the stench from the holds being almost insupportable, and totally incompatible with health. It may also be added that, on subsequently clearing her out, the corn and yams with which she was freighted were found to be in a state of decomposition.

"Shortly after her arrival, tents were erected on shore, and the whole of the crew were landed and placed in them; the sick being separated from the healthy. This precaution was unnecessary, or at least without the desired effect, as the few remaining Europeans and three Africans were almost immediately added to the list; making a total of thirty-nine, of whom twenty-eight recovered and returned to duty, three were invalided and sent to England, and eight died.

"The fever in this instance appears to have displayed all the usual characteristics of the common remittent or yellow fever;—yellowness of skin, bleeding of the gums, and black vomit. It is to be regretted that there are not any means of obtaining information relative to the first appearance of the disease, or of its progress prior to her arrival at Ascension on the 30th of January, in the deplorable condition previously described. The first assistant-surgeon who took charge of her, after her arrival, does not allude to the question of contagion; the other who succeeded him on the 16th of February states that, from the information he had received, he was led to regard the disease as decidedly contagious. The sick were admitted to pratique on the 1st of March." P. 122.

With respect to the "Forester," all that we learn is that, "in the early part of the year, and again in May, fever of a most virulent character assailed the ship's company at Sierra Leone, and nineteen fell victims, being upwards of a third part of the whole: amongst them was the assistant surgeon. There is therefore not any history of the disease until the 20th of June, when two-thirds of the crew only were in existence, and in a state of convalescence."

Besides these vessels, the "Curlew," the "Fair Rosamond," and the "Waterwitch" suffered severely in the first-half of this year, 1838. The history of the last-named ship deserves particular notice.

"The 'Waterwitch' was employed cruising on the north part of the station, the crew being healthy until she arrived at Ascension in April, when numerous cases of diarrhœa occurred, supposed to have been occasioned by the water of that island having been drunk while in a turbid state. Its progress was immediately checked by abstaining from the cause. About this time a malignant fever prevailed among the inhabitants on shore, but there was not any sickness on board the Waterwitch up to the 3rd of May, when she left for the coast of Africa. On that day one case of malignant fever occurred; and again on the 13th, ten days after being at sea, there were four more added to the list, all resembling the first, but presenting more decidedly the characteristics of true yellow fever. From this time the malady continued its ravages until the 4th of June; within that period no less than sixty had been attacked, and fifteen died. The crew consisted of fifty-two Europeans and eighteen native Africans; only three of the white men escaped. Many of the Africans also suffered, but their attacks were comparatively mild, and not attended with danger.

"There was not any possibility of separating the sick from the healthy: but ventilation and cleansing were had recourse to as far as circumstances would admit, although unfortunately this was next to an impossibility, so great was the quantity of provisions and other stores taken on board at Ascension for the use of the squadron. The state of the weather, together with the locality (the Bight of Biafra), where the rains had just commenced, also tended to aggravate the disease. As the vessel approached the coast, the number of cases increased with such rapidity, that there were at one time twenty-three fresh attacks within the space of three days, leaving only five white men to do duty on deck, although from the 4th to the 6th of June there had been only three!

"The fever was characterised by remissions, yellowness of skin, black vomit, black urine, hæmorrhage from the nostrils, throat and mouth, and vomiting of blood. There were several recoveries after hæmorrhage from the mouth, throat, and nostrils had taken place, but there were not any after black vomit had occurred." P. 128.

The "malignant fever among the inhabitants on shore," here alluded to, appears to have broken out in the latter part of March, after heavy rains on the 16th and 17th of that month, preceded by some years (?) of dry weather. "Among the people of the island, there prevailed an opinion that the disease was imported by the vessels from the coast"—viz. the Ætna, the Forester, and the Bonetta. The surgeon, however, of the hospital at Ascension seems to have been of a different opinion, and to have regarded the disease as of local origin. The town was, at the period of the invasion, in a most offensive state, in consequence of the collection of stagnant putrid water in many parts of it; but whether we can admit the sufficiency of this as the real and primary cause of the epidemic, and quite independently of the arrival of sickly vessels from the African coast, which had been more than usually unhealthy during the season, we shall leave to our readers to judge for themselves, more especially as Dr. Bryson does not think fit to express his own sentiments upon the question.

There is nothing in the medical history of the following 5 years (1839—43) that calls for particular notice. At no time, does malignant fever appear to have prevailed epidemically on board any of the squadron, or at any of the settlements.* We shall therefore pass to the year 1844, during which there was certainly more than the average amount of sickness in several of the ships on the station. In the early part of the year, the "Hydra" (which had been cruising off the coast to the South of the Line, and had also been up the Congo river) suffered not only from remittent fever, but also severely from diarrhœa; no fewer than seventy-two cases of this disease having occurred. Upon this point, Dr. Bryson makes the important remark that "this has been a frequent precursor of severe epidemic attacks of fever upon the station; it was so in the "Eclair," and in many other instances heretofore noticed in these remarks; there is reason therefore to suppose that it is but a different effect of the same general morbific cause. It does not appear, however, that the diarrhœal affection has any effect in warding off an attack of fever; it is in fact more of the nature of a predisponent than of a prophylactic; while by lowering the general tone of health, it renders the constitution less capable of withstanding the shock of the disease afterwards." Towards the end of the year, fever again made its appearance on board the Hydra, and then seemed to assume two forms; "'one being mild, and the other malignant;' the former was the more general, and occurred among men who had not been out of the ship, particularly amongst the stokers; the latter, few in number, occurred in the gig's crew, who were exposed to concentrated malaria for a period of forty-eight hours in the river Sherbro."

During the last three months of the year, the "Growler" steamer had 63 cases of fever, 21 of febrile catarrh, and 11 of dysentery—"an amount of disease that clearly enough indicates the pestilential nature of the locality," Sierra Leone and the adjoining coast. Twenty fresh cases of fever occurred in the course of Jan. and Feb. 1845: three of these terminated fatally. In March, she went to the Cape de Verde islands to recruit the health of her crew, returned again to her old station, where she remained till the following August, and then returned to England.

The "Lily" and the "Penelope" do not seem to have suffered more than usual during this year, 1845; but we find that on board the "Styx," six days after leaving Fernando Po where the crew had worked hard in coaling and watering the ship, fever of a malignant type made its appearance. From the 23rd of November to the 3rd of December, thirteen cases in all occurred; and of these, five proved fatal—one on the third day, three

* It may be of importance to know that, during the interval mentioned, the men were much less engaged in the harassing and dangerous duties of boat-service than they had ever been before.

on the seventh, and one on the ninth. " During the early period of the disease, the exacerbations and remissions were remarkably distinct, there being a good day and a bad one alternately; on the good day there was nearly a total remission of febrile symptoms; but, as the fever advanced, and the vital energies declined, the remissions became more irregular, the skin being sometimes dry and harsh, at other times covered with a cold clammy perspiration; the thirst was then great, the tongue became cleaner and sometimes red and shining. One of the fatal cases occurred in a Krooman; in him the disease presented a very aggravated form, with early and restless delirium."

The medical history of the "Eclair," from the time of her leaving England in Nov. 1844 to her disastrous return in September of the following year, is traced with considerable minuteness by Dr. Bryson. As a matter of course, we have no occasion to follow him in this narrative. We shall, therefore, merely mark one or two topics that will serve to complete the lengthened details which we have already given, first in the number of this Journal for July 1846, and subsequently in that for July of the present year, when we brought the instructive report of Dr. McWilliam under the attention of the reader.

Between the 8th of March and the 3rd of April, a number of cases of Diarrhœa occurred on board. As already stated, this is a frequent precursor of the invasion of malignant fever on the coast of Africa.

The first cases of fever were observed in the men who had been employed in the boat expeditions in the Sherbro river. Up to the 15th of June, fourteen cases in all had occurred; and, of these, no fewer than nine had proved fatal; seven among those (26 or 28 in number) who had been in the boat expeditions, and two among those who had not been out of the ship. From this circumstance Dr. Bryson infers "that the disease was contracted from local causes exterior to the ship; for although two cases occurred in persons who had not been out of her, still, from her close proximity to the land, the whole crew must have been more or less exposed to the same malarious emanations as the men employed in the boats, although perhaps in a less concentrated form, while, not having suffered from privation and fatigue, they were not so susceptible of the disease."

Notwithstanding the amount of sickness already experienced, it appears that the expeditions in the boats were not discontinued until the 2nd of July, when the steamer sailed for Sierra Leone, where she arrived on the 4th, "the crew, comparatively speaking, healthy, and the last remaining cases of fever advancing favourably towards convalescence." The subsequent stay there for three weeks in one of the most unhealthy months of the year; the cleaning out of the hold of the "Albert," with the irregularities and excesses then committed; the unfortunate permission granted to the men to go on shore, few returning at sunset, as ordered; the subsequent exposure of part of the men in painting and refitting the other steamer—these are circumstances that are well known, and will not fail to be taken into account in examining the history of the Eclair epidemy. Not a word is said by Dr. Bryson as to the time when the first case of "black-vomit" occurred among the fever patients; although (it will be remembered) Sir William Pym took it upon himself to fix the date with precision! We need scarcely say that he disapproves, in the strongest terms, of the delay on the part of Sir William to have the crew immediately removed from the vessel on her arrival at the Motherbank. On this point there can be but one opinion, and that opinion is against the measures that were taken.

We do not remember having seen the following account of the fever on board, as it existed when Mr. Bernard joined the vessel at Madeira: Mr. B., in his first report, says:—

"The cases which presented during the above period were characterized by intense frontal head-ache, and a sensation of weight over the eyes, severe pain across the loins, and in some cases there were general pains; the tongue loaded with a white mucus in the centre, leaving the edges and apex of a bright red, its substance firm; thirst urgent, the skin hot and dry, bowels constipated, the pulse small and without any hardness. In the course of about six hours, vomiting of a greenish yellow fluid took place, accompanied with pain in the epigastrium, or across the chest. The vomiting then became continuous. The dejections procured by purgatives or enemata were dark and fetid. On the evening of the second day brownish flocculi might be detected in the fluid vomited, which increased on the third day, by which time a sinking of the pulse was observable, with coldness of the extremities, and sometimes low delirium, whilst at others there was a wildness of manner, and a disinclination or absolute refusal to take either food, drink, or medicine. The tongue then became of a bright red colour; in some it was quite moist, but generally dry. From this state none rallied. Such were the prominent symptoms, although the latter sometimes did not occur until the fourth or fifth day. The Kroomen, who are still on board, and have been employed in attendance on the sick, have not suffered." P. 191.

There is no mention here of any remissions, or tendency to their occurrence; and Dr. Bryson subsequently says that the fever was "of a typhoid character."* In conclusion, Dr. B. points to the close similarity between the cases of the Eclair and of the Bann, as we had previously done in our article of last year. His words are—"Both vessels contracted the disease at Sierra Leone, and apparently from the same cause or causes, and under similar circumstances. In both vessels, in the course of a few weeks it assumed an epidemic character, if it did not acquire contagious properties; the one vessel proceeded to the barren rocky island of Ascension, a few degrees to the south of the equator, where a disease of the same character made its appearance amongst the inhabitants, and committed great ravages; the other proceeded to the nearly equally barren island of Bona Vista, a few degrees to the north of the equator, where in like manner a disease a short time afterwards broke out, and raged with equal severity."

From the tabular account given by Dr. Bryson of the total loss sustained by each vessel of the squadron on the African Coast, during the year 1845, it appears that, while the Eclair lost no fewer than 74 men from fever, the greatest mortality from this cause on board any other ship did not exceed 3! The following table,—exhibiting the annual mean strength, and the number of deaths from disease, from accident, and from all causes,

---

* The following statement, which, as far as we know, has not been made known before, deserves to be noticed:

"The holds of the Eclair it was supposed had been made perfectly clean, while her crew were disembarked at Bona Vista; but there was afterwards found, when she was re-commissioned, a large collection of mud, fully three inches in depth, upon that portion of her bottom occupied by the boilers and machinery, which apparently had not been disturbed for a long time." P. 223.

between the years 1825 and 1845, both years inclusive—serves well to point out how pre-eminently sickly some seasons were above others.

| Year. | Annual Mean Force. | Deaths. From Disease. | Deaths. From Accident. | Total Deaths from all causes. |
|---|---|---|---|---|
| 1825 | 663 | 41 | 7 | 48 |
| 1826 | 1043 | 57 | 6 | 63 |
| 1827 | 955 | 40 | 4 | 44 |
| 1828 | 958 | 81 | 3 | 84 |
| 1829 | 792 | 202 | 2 | 204 |
| 1830 | 667 | 72 | 4 | 76 |
| 1831 | 785 | 22 | 3 | 25 |
| 1832 | 512 | 18 | 3 | 21 |
| 1833 | 562 | 12 | 10 | 22 |
| 1834 | 620 | 18 | 8 | 26 |
| 1835 | 815 | 19 | 3 | 22 |
| 1836 | 965 | 16 | 4 | 20 |
| 1837 | 815 | 105 | 4 | 109 |
| 1838 | 885 | 115 | 3 | 118 |
| 1839 | 790 | 55 | 5 | 60 |
| 1840 | 855 | 32 | 3 | 35 |
| 1841 | 1070 | 68 | 17 | 85 |
| 1842 | 1330 | 43 | 29 | 72 |
| 1843 | 1267 | 23 | 4 | 27 |
| 1844 | 1715 | 43 | 6 | 99 |
| 1845 | 2540 | 121 | 7 | 128 |
| | 20,604 | 1203 | 135 | 1338 |

"Total mean force for twenty-one years 20,604; ratio of mortality per 1000 of mean force, 58·4; ditto from all causes, 64·9." P. 177.

"It would thus appear," adds our author, "that the annual ratio of mortality from disease alone on the African station for a period of twenty-one years, was 54·4 per 1000 of the mean force employed. The fatal nature of the climate, however, becomes more apparent when placed in juxta-position with the mortality on other stations, to wit:—

| | |
|---|---|
| South America . . . . | 7.7 |
| Mediterranean . . . . | 9.3 |
| Home . . . . . | 9.8 |
| East Indies . : . . | 15.1 |
| West Indies . . . . | 18.1 |
| Coast of Africa . . . | 54.4 |

"It is proper, nevertheless, to observe that nearly one-half of this proportional amount resulted from epidemic fever alone, which was confined to a few vessels of the squadron during the years 1828-9 and 30; again in 1837-8, and 39, and in the Eclair in 1845. Deducting the loss from epidemic fevers, therefore, the ratio of mortality from all other classes of disease on the station will be about 20·0 per 1000 of the mean force annually; this, however, can give no adequate idea of the permanent loss of health, which is assumed to be great. Still, from these and other data, it seems fair to deduce that if boat-service were in some degree restricted; if prize crews were not permitted to land at Sierra

Leone, and if all vessels contracting epidemic disease were to leave the station, and proceed directly to a colder climate, the ratio of mortality, and the permanent loss of health one year with another would be reduced at least nearly one-half." P. 178.

And here we must draw our narrative to a close. With a few remarks on the general history of that disease, which, as we have seen, is every now and then apt to prove so terrible a scourge to our brave seamen in their perilous duties—would that success commensurate with the peril incurred, not to say with the sacred justice of the cause, might reasonably be expected !—off the fatal coast of Africa, we shall conclude.

From the following passage it will be seen that the views of Dr. Bryson, respecting the nature of true Yellow Fever, and also upon the important question of its communicability or power of infectious propagation, correspond very exactly with those which we endeavoured to enforce in our last number. He first alludes to the confusion that has been introduced into the nosological returns of the navy, by the adoption of different names applied to the same disease by different medical officers.

"The bilious remittent of one person was found to be the climatorial of another; the endemic of a third was the typhus icterodes of a fourth; the adjectives ardent, yellow, congestive, inflammatory, had all been used in describing the same disease. A more simple phraseology was therefore, unless under peculiar circumstances, deemed advisable. The character of these fevers, in fact, is such that the synochal of one day may become a remittent on the next, and probably ere long terminate in an intermittent; the ephemeral of little force may suddenly become one of high vascular action; or at the same time, but in a different subject, pass rapidly through the stage of excitement, and at once enter upon the *typhoid;* while that which invades with great intensity of action may frequently be of ephemeral existence only. It is therefore obvious that it is not until the fever approaches its termination that it can be brought under any one of the previous heads; consequently in a practical point of view such visionary distinctions are of little or no importance." P. 250.

He then subjoins the important remark :

"The fevers of Africa, strictly speaking, are only divisible into two kinds; namely into the remittent and intermittent. The former, however, may be subdivided into the endemic, epidemic, and contagious; but as either of the former, as in the Bann, may be converted into the latter by improper ventilation, the depressing passions, and physical prostration, and as it—the contagious—does not originate or even exist for any length of time except under these conditions, the subdivision is again reduced to two heads—the endemic and epidemic, both of which are remittent, and both generally, according to their persistence, attended with more or less yellowness of the skin, and occasionally in the more severe cases with black-vomit. It becomes a question if the latter be not an aggravated type of the former, in consequence of the more general prevalence of a common exciting cause. Still, from its uncertain modes of invasion at distant periods; from its apparent restriction to certain bounds; and from its greater severity, the appellative distinction, at least until the subject is better understood, remains strictly warrantable." P. 250.

Notwithstanding the doubt expressed in the last sentence, the fair and legitimate deduction from all the statements of this volume appears to us to be, that the malignant or pestilential malady, known as the true or genuine Yellow Fever is but an aggravated and epidemic form of the ordinary and endemic fever of the coast; this having become—from causes which we but very imperfectly understand*—more of a continued type, and being then accompanied with symptoms that are clearly indicative of a dissolved or diffluent state of the blood. That Dr. Bryson regards it as of the nature of Typhus, would appear from his using such expressions as the following in reference to the disease as it existed on board different ships :—" the typhoid stage of yellow fever;" the fever was of " the nature of *typhus icterodes* ;" it was of " a typhoid character," &c. The deeply altered state of the circulating fluid is alluded to in various passages. For example, we read in one that " the blood, when drawn, is sometimes described as being very dark-coloured, and, when allowed to stand, was loose in its texture, neither shewing a buffy coat, nor separating into serum and crassamentum." Many similar statements are made in other parts of the volume.

We need scarcely say that not the slightest countenance is given by anything which occurs in Dr. Bryson's Report to the doctrine of the yellow fever being a "nova pestis"; an idea originally suggested by Chisholm, and still, most strangely, believed in by Sir William Pym.

We have seen that our author recognises the occasional and contingent infectiousness of the disease, by direct effluvia from the body of the sick. No warrant, however, is to be found in his work for the belief that it was ever conveyed or transmitted by *fomites*. The only instance, in which reference is made to this matter, occurs in the description of the outbreak of the pestilence at Ascension in 1838, soon after the arrival of the "Ætna," the " Forester," and the "Bonetta," as mentioned in a preceding page. It was asserted, by certain of the inhabitants of the island, that the infection had been introduced by the clothes of the deceased officers having been sold to some of the people on shore. The following statement will remind the reader of Dr. McWilliam's evidence about the washerwomen at Bona Vista, as recorded in our last number.

" Sergeant Warren most unquestionably made the most extensive purchases, and his wife took in washing; they both certainly died of the disease, but neither of them was attacked until a late period of the sickness, and both had been attending day and night in the crowded houses of their sick neighbours. Mrs. Scarisbrook was also an extensive purchaser; she was, however, the very last woman attacked, and her husband entirely escaped the disease. George Downes purchased some wearing apparel, which he took to his house, where he had a wife and three children; he has worn a thick watch coat that belonged to the late Lieutenant ——— (who died of fever) on his night watch ever-since, yet neither he nor any of his family have been attacked. They probably owe their immunity simply and solely to their house being situated at a considerable distance to windward of the squares, and at an elevation of several hundred feet. In short, many who were purchasers escaped the disease, while others who did not make any purchases were attacked." P. 133.

Had our space permitted, we should gladly have followed Dr. Bryson in his excellent remarks on the important question of the Treatment of

---

* However true this remark may be, as applied to the disease when prevailing *epidemically*, it is always to be remembered that, in all seasons, the ordinary coast fever will sometimes put on all the phenomena of the worst malignancy, when the men have been allowed to sleep on shore, and have been committing great excesses. These occasional cases, however, never exhibit any tendency to spread.

Yellow Fever. We very strongly recommend every tropical practitioner to study them with attention. It is quite obvious that no inconsiderable amount of mischief has, not unfrequently, been done by the rash and injudicious adoption of over-active measures—the heroic method of treatment, as it has been absurdly termed—in a true *blood-disease*, such as the African pestilence unquestionably is. Does not the following statement read a useful lesson to the ultraist practitioner? At the time when the "Eden" was suffering most severely from the fever, and her medical officer was one of the victims, "the office of surgeon (there being no assistant) was then assumed by the captain, whose extensive knowledge and long experience of the African climate and diseases, rendered him peculiarly fitted to perform its duties. His treatment of this formidable disease, it is stated, was simple, but more successful than any that had hitherto been adopted. Having witnessed the frequent and fatal result of 'energetic treatment,' he had imbibed a kind of horror of bleeding, and, at the same time, a predilection in favour of mild measures, probably from observing the greater success that attended the simple means employed by the natives and resident Europeans. The abstraction of blood did not therefore form any part of his treatment. He commenced with some brisk purgative, and after its operation, patiently waited for a remission of the symptoms, when he exhibited quinine, and continued its use, until the patient got well; omitting to give it, however, if a paroxysm of fever intervened. When diarrhœa supervened during convalescence, which was not unusual, he gave calomel until ptyalism was fully established; after which the patient generally recovered rapidly."

Dr. Bryson dwells with marked emphasis on the pernicious results that have, in many instances, flowed from the too common practice of pushing the mercurial treatment to a most extravagant length. Even when salivation has been induced, and the system has therefore been brought under the influence of the medicine, no benefit has followed.

There seems to be no difference of opinion as to the propriety, nay the necessity, of administering the bark or quinine freely, whenever there is evidence of the accession of the apyretic stage. [Does not this circumstance alone testify most strongly as to the real *nature* of the disease?] But, alas! in very many cases, there is no distinct cessation, scarcely an abatement, of the pyrexial symptoms, and all medication is utterly profitless. Hence the experienced naval surgeon will often have his mind much more intent upon precautionary and prophylactic, than upon (what are called) curative, measures; for he knows well that, while much may frequently be done, by the timely adoption of appropriate hygienic means, for the prevention and arrest of malignant fever on board a ship, too often all his professional skill is of little or no avail when the enemy has once shewn itself among the crew. And here we must not omit to mention that several instances are recorded, in the Report now before us, of fever, if not arising directly from, at least being strikingly aggravated both in intensity and frequency by, causes *within* the vessel itself. The reader has only to refer to pages 224, 228, 229, and 230 to be convinced of this. It will be useful to notice a judicious caution, which Dr. Bryson gives to naval officers, touching this subject:

"It would be well to avoid, under all ordinary circumstances, attempting to clear out a vessel on the spot where the disease originated; more particularly if there be reason to suppose it has arisen from a foul state of the holds, for by opening and disturbing the various matters contained in them, the cause must necessarily be let loose upon the men with increased force, while the latter, in a state of fear and despondency, being aware of the danger, are rendered more obnoxious to its influence, and brought, by the nature of their duties, more immediately within its sphere of action. It will be time enough, after the entire cessation of the disease, when by a change of climate and diet the general health of the ship's company has become invigorated, and when confidence has been restored, to commence the work of expurgation in the vessel. It is by no means uncommon for an epidemic to become aggravated by opening up the holds of a ship within the tropics." P. 229.

In concluding our notice of this interesting Report, we would earnestly urge upon all young surgeons of our ships of war the great importance of their keeping an *exact* and *regular* account of the health of the crew, and of the origin, progress and decline of fevers and other constitutional diseases on board, with remarks on the influence of all causes, whether these be *ab extra* or *ab intra*, whether connected with the nature of the climate, the season of the year, the character of the men's occupations, or in their food or the state of the ship itself, &c. &c. To be of any decided utility, such observations must be continued not for a few weeks or months only, but for several years; for it is in this manner alone that we can ever reasonably hope to understand something more than we yet do of the true history of certain Epidemic diseases ;—which, be it remembered, are only of occasional and of unforeseen occurrence, and not of an annual or constant prevalence. We say "unforeseen," because, as yet, there are scarcely any data to enable a medical man to form even a rational conjecture on the subject. But is it so utterly inconsistent with our knowledge of the operations of nature in respect of climatorial and other atmospheric conditions, that we must never entertain the hope that a discovery may yet be made by some carefully-observing and deep-reflecting medical philosopher to enable us to anticipate, in a measure at least, the recurrence of those fearful visitations of wide-spread disease, to which the term "pestilential" is usually applied? We think not. At all events, nothing can even be expected to be done in this direction, unless there has been previously accumulated a large store of accurate facts and observations, perseveringly registered during a succession of many years. Hitherto this has been very imperfectly done; and the humiliating reflection abides with us, that we are just about as ignorant of almost everything appertaining to the history of Yellow Fever as our forefathers were.

